# Intestinal regulation of *suppression of tumorigenicity 14 (ST14)* and *serine peptidase inhibitor, Kunitz type -1 (SPINT1)* by transcription factor CDX2

**DOI:** 10.1038/s41598-018-30216-z

**Published:** 2018-08-07

**Authors:** E. Thomas Danielsen, Anders Krüger Olsen, Mehmet Coskun, Annika W. Nonboe, Sylvester Larsen, Katja Dahlgaard, Eric Paul Bennett, Cathy Mitchelmore, Lotte Katrine Vogel, Jesper Thorvald Troelsen

**Affiliations:** 1 Department of Science and Environment, Roskilde University, Roskilde, Denmark; 2 Institute of Cellular and Molecular Medicine, the Panum Institute, University of Copenhagen, Copenhagen, Denmark; 3 Department of Gastroenterology, University of Copenhagen, DK-2730 Herlev, Denmark; 4 Department of Clinical Immunology, Naestved Hospital, Naestved, Region Zealand Denmark; 5 Copenhagen Center for Glycomics, Department of Odontology, Faculty of Health Sciences, University of Copenhagen, Copenhagen, Denmark

## Abstract

The type II membrane-anchored serine protease, matriptase, encoded by *suppression of tumorgenicity-14* (*ST14*) regulates the integrity of the intestinal epithelial barrier in concert with its inhibitor, HAI-1 encoded by *serine peptidase inhibitor, Kunitz type -1* (*SPINT1*). The balance of the protease/inhibitor gene expression ratio is vital in preventing the oncogenic potential of matriptase. The intestinal cell lineage is regulated by a transcriptional regulatory network where the tumor suppressor, Caudal homeobox 2 (CDX2) is considered to be an intestinal master transcription factor. In this study, we show that CDX2 has a dual function in regulating both *ST14* and *SPINT1*, gene expression in intestinal cells. We find that CDX2 is not required for the basal *ST14* and *SPINT1* gene expression; however changes in CDX2 expression affects the *ST14*/*SPINT1* mRNA ratio. Exploring CDX2 ChIP-seq data from intestinal cell lines, we identified genomic CDX2-enriched enhancer elements for both *ST14* and *SPINT1*, which regulate their corresponding gene promoter activity. We show that CDX2 displays both repressive and enhancing regulatory abilities in a cell specific manner. Together, these data reveal new insight into transcriptional mechanisms controlling the intestinal matriptase/inhibitor balance.

## Introduction

The intestinal epithelium represents one of the most significant permeability barriers for exposure to environmental toxins and microorganisms^1^. Dysfunction of barrier integrity can contribute to local and systemic diseases including inflammatory bowel disease, allergic disorders and malignancies^1,2^.

Evidence from the last decade has demonstrated the crucial importance of the serine protease matriptase in the generation and maintenance of epithelial integrity^3–6^. Matriptase (also denoted MT-SP1, epithin, TADG-15 and SNC19) is a type II transmembrane serine protease encoded by the gene *suppression of tumorgenicity-14* (*ST14*), and is expressed in most epithelia^7–10^. The *ST14* gene has been established as a critical tumor-suppressor gene in the gastrointestinal tract, as well as a suppressor of colitis and colitis-associated colon carcinogenesis in mice^5,11^. However, it is currently unknown how the *ST14* gene is involved in maintaining epithelial barrier integrity, and in suppressing intestinal carcinogenesis.

Matriptase is known to be kept under strict post-translational control by its two inhibitors, HAI-1 and HAI-2, encoded by the genes *serine peptidase inhibitor Kunitz type -1 and -2* (*SPINT1* and *SPINT2*)^9,12–14^. Evidence suggests that matriptase becomes highly oncogenic, when not kept under strict post-translational regulation by either HAI-1 and/or HAI-2, as deregulated matriptase has been shown to cause squamous cell carcinoma formation in transgenic mice overexpressing only wild-type matriptase in the epidermis, while a simultaneous increase in either the HAI-1 and/or HAI-2 expression completely reverses the oncogenic potential caused by matriptase overexpression^15–17^. Furthermore, an increase in the matriptase/HAI-1 protein ratio has been identified in late-stage ovarian tumors^18^, as well as for the mRNA ratio in colorectal cancer adenomas and carcinomas^19^, proposing that matriptase and/or HAI-1 may be deregulated during tumor progression.

While the regulation of matriptase at the protein level has been well studied, less is known about the transcriptional regulation. The critical importance of the protease/inhibitor mRNA ratio found in the intestine suggests that the expression ratio of *ST14* and *SPINT1* might be co-regulated at the transcriptional level by intestine-specific transcription factors.

The intestinal epithelium-specific regulatory network of transcription factors has been well studied^20–22^, and the *caudal*-related homeobox (CDX2) transcription factor, CDX2, has been revealed as an intestinal master transcription factor. Studies in mice have shown that Cdx2 plays a vital role during intestinal development, differentiation, and during adult tissue homeostasis^23,24^. Loss of CDX2 affects the tissue expression pattern and morphology in both the adult small intestine and the colon^24,25^. Cultured *in vitro* 3D-grown mouse-derived small intestinal organoids have shown that intestinal stem cells lacking CDX2 fail to commit to the intestinal differentiation program and instead turn into a more gastric-like lineage^26^. Several clinical studies have observed dysregulated CDX2 levels in colorectal cancer^27^, and reduced levels of the transcription factor have been reported to be a prognostic biomarker for stage II and III colon cancer^28^ and metastatic colon cancer^29^. Moreover, studies have demonstrated that overexpression of CDX2 has an inhibitory effect on colon cancer growth in *in vitro* experiments and in tumor-transplantation studies performed in mice^30,31^.

In this study, we report that the *ST14* and *SPINT1* basal gene expression is not dependent on CDX2 in intestinal cells; however, induced expression of CDX2 affects the *ST14/SPINT1* balance. We have identified and characterized functional intestine-specific transcriptional DNA regulatory enhancers for both the *ST14* and the *SPINT1* genes, and shown that CDX2 binding is enriched within these enhancers, using chromatin immunoprecipitation, as well as described the specific binding sites, using gel shift assays. Collectively, these results provide evidence that the *ST14* and the *SPINT1* enhancers functionally activate their corresponding promoter activity, in an intestinal- and CDX2-regulated manner. Thus, we suggest that the intestine-specific co-expression of matriptase and its inhibitor HAI-1 involves transcriptional regulation by the transcription factor CDX2.

## Results

### CDX2 stimulates *ST14* gene expression while repressing *SPINT1* gene expression in intestinal epithelial cells

**Figure 1:**
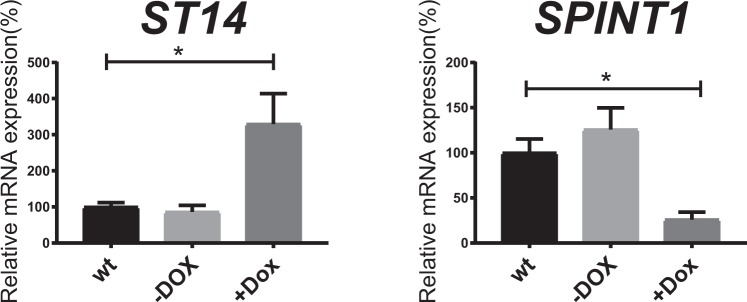
CDX2 stimulates *ST14* mRNA expression and inhibits *SPINT1* mRNA expression in intestinal epithelial cells. In the absence of doxycycline (−Dox), the LS174T intestinal cell line with Tet-On inducible system to control CDX2 expression has knocked-out the endogenous CDX2 genomic locus using a Tet3G transactivator element (described in^32^). Treatment with doxycycline (+Dox) stimulates ectopic CDX2 expression from the Dox-inducible cassette. Experiments are compared to wild-type (wt) LS174T cells harboring no Tet-On system. Relative gene expression of *ST14* and *SPINT1* were normalized to *β-Actin* mRNA levels. Data are expressed as mean values ± S.E.M (n = 4), *P < 0.05 (one-way ANOVA analysis).

To explore whether the intestinal master transcription factor CDX2 influences the intestinal gene expression of matriptase and its inhibitors HAI-1, their gene expressions were investigated using the LS174T colorectal cell line with a conditional CDX2 knock-out/knock-in system, as recently described^32^. The LS174T cells, harboring trans-activator elements (TET3G) and the PrIITE system, were engineered to disrupt the endogenous CDX2 locus, and upon stimulation by doxycycline, to induce ectopic expression of a codon-optimized CDX2 construct. The analyses showed that loss of CDX2 (- Dox) did not have an impact on either *ST14* or *SPINT1* mRNA expression, as compared to wild-type LS174T cells (wt), suggesting that their basal gene expression is not maintained by CDX2, thus showing CDX2 independence (Fig. 1). However, doxycycline-induced CDX2 expression (+Dox) significantly increased *ST14* mRNA expression compared to wt and -Dox, whereas CDX2 significantly reduced *SPINT1* mRNA expression compared to -Dox (Fig. 1), suggesting that CDX2 has the ability to modulate the *ST14*/*SPINT1* gene expression ratio in intestinal cells.

### Identification of CDX2-regulatory enhancer sites in the *ST14* and *SPINT1* genes in intestinal epithelial cells

We next investigated whether CDX2 regulates gene expression of *ST14* and *SPINT1*, the genes encoding matriptase and HAI-1 respectively, in a direct or an indirect manner. For this, we analyzed sets of previously obtained genomic intestinal CDX2 ChIP-seq data, to uncover any potential CDX2-regulatory sites near the genomic locus of the *ST14* and the *SPINT1* genes. Previous data of CDX2 ChIP-seq tracks from both LS174T cells and Caco-2 cells, human colorectal adenocarcinoma-derived cell lines which have been used as a model for intestinal epithelium, were analyzed^21,32^. Caco-2 cells have the unique ability to spontaneously differentiate into polarized columnar epithelial cells with intestinal characteristics^33^, and are therefore often used as a model to study the regulation of intestinal genes. The Caco-2 cells have previously been shown to express both the *ST14* and the *SPINT1* genes endogenously^34^.

**Figure 2:**
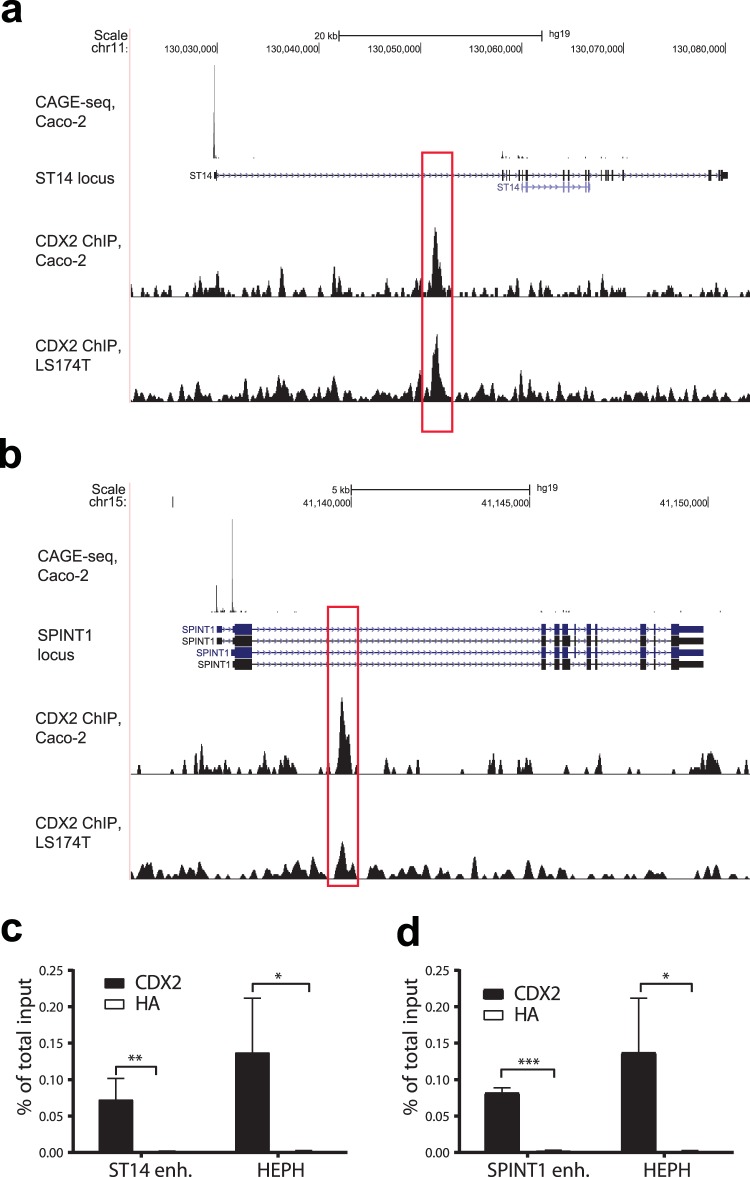
Identification of CDX2-regulatory sites for the *ST14* and *SPINT1* genes in Caco-2 cells. The genomic overview of *ST14* in (**a**) and for *SPINT1* in (**b**) are obtained from the UCSC Genome Browser (hg19)^74^. CAGE-seq tracks shows 5′ end of mRNA transcription in Caco-2 cells. CDX2 ChIP-seq tracks in Caco-2 and LS174T cells are derived from previous publications^21,32^. Marked in red box is the identified potential *ST14* enhancer was located in the first intron with position +21579 to +22159 (580 bp) relative to the transcriptional start site and the identified *SPINT1* enhancer was positioned in the first intron, nt +3331 to +3863 (532 bp) relative to the transcriptional start site. CDX2 and HA-ChIP PCR on confluent Caco-2 cells verified the CDX2 enrichment of the *ST14* and *SPINT1* enhancer regions in (**c** and **d**) respectively. HA-enrichment serves as a non-specific negative control. *HEPH* represents the *Hephastatin* promoter, a known CDX2 target gene. Immunoprecipitated DNA are presented as percentage of total DNA input and represent the mean with S.D. (n = 3–4). CDX2-binding enrichments are statistically significant from the negative HA-enriched control samples, *P < 0.05 (Student’s T-test).

When analyzing the ChIP-seq tracks, no convincing CDX2 binding peaks were found in the nearby promoter regions of the *ST14* and the *SPINT1* gene. However, the tracks revealed a distinct CDX2-binding peak within the first intron of each gene, between position +21579 to +22159 relative to the *ST14* transcriptional start site (TSS) and between +3331 to +3863 relative to the *SPINT1* TSS (Fig. 2a and b). Additionally, the Caco-2 ChIP-seq tracks revealed that these distinct CDX2-binding peak regions were covered by H3K4me2 marks (Figure S1 and S2), suggesting that these genomic sites have an open chromatin structure, making them possible active cis-regulatory enhancer elements in intestinal cells. These CDX2-bound regions within the *ST14* and *SPINT1* genomic locus were chosen to be characterized and investigated for their potential role of being enhancers for the gene promoters in intestinal cells.

The CDX2 signal enrichments from the previously performed ChIP-seq data were confirmed with a new CDX2 enrichment ChIP analysis on Caco-2 cells, using ChIP-qPCR with primers covering the enhancer regions. Primers covering the promoter for *HEPH*, encoding an ion transport protein that is regulated by CDX2 in the intestinal epithelium^35^, were included in the qPCR as a positive control for CDX2-enrichment as previously demonstrated^21,36^. Both the *ST14* and *SPINT1* enhancer regions were significantly enriched in the CDX2 ChIP, as compared to negative control HA ChIP (Fig. 2c and d). The intensity of CDX2-binding enrichment of the *ST14* and *SPINT1* enhancer sites were comparable to the binding enrichment of the HEPH target region, suggesting that both the *ST14* and the *SPINT1* genes contain direct binding sites for CDX2.

Altogether, these results indicate that CDX2 binds to putative regulatory sequences in *ST14* and *SPINT1* genes site, which is marked as a potential epigenetically active enhancer region for both *ST14* and *SPINT1* in intestinal cells.

### *ST14* and *SPINT1* enhancers are functional in intestinal cells

**Figure 3:**
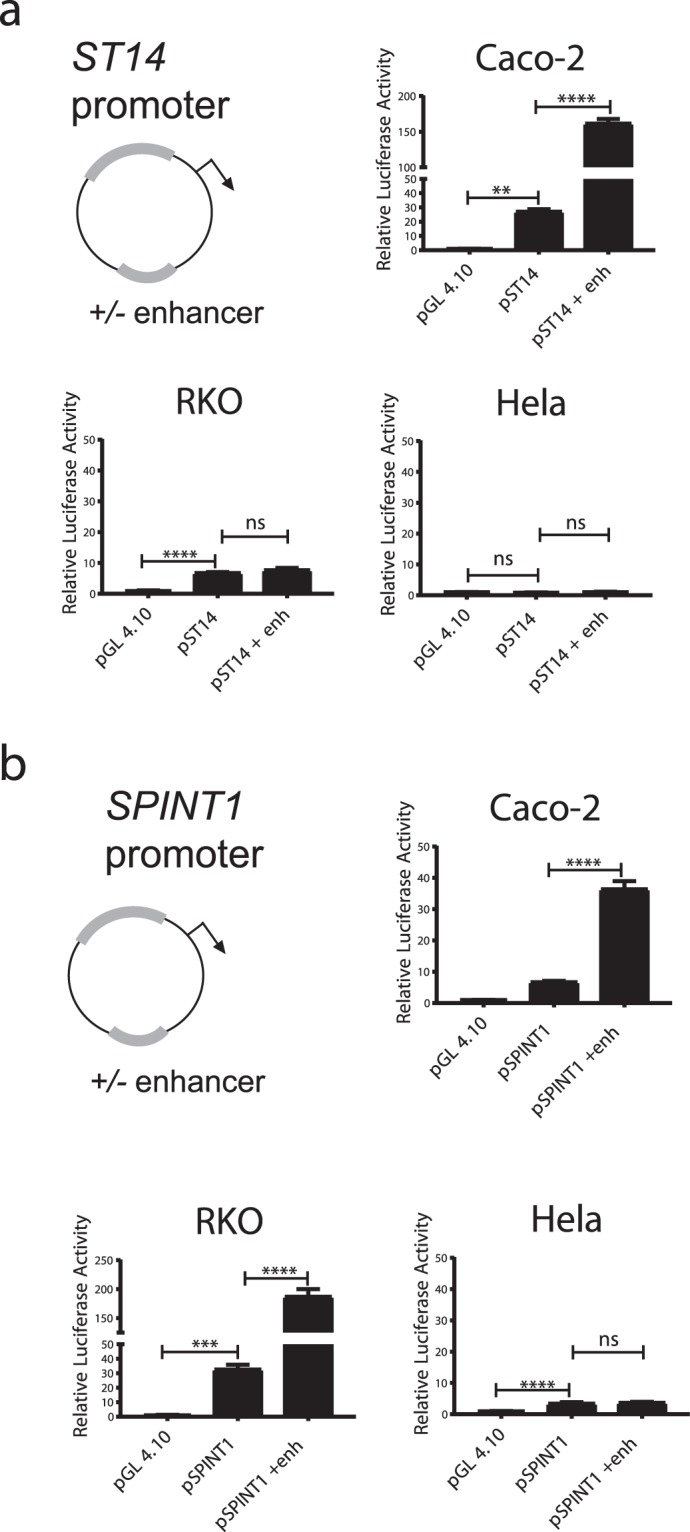
*ST14* and *SPINT1* promotors are regulated by enhancer activity in colorectal cells. Luciferase reporter plasmids containing the *ST14* (**a**) or *SPINT1* (**a**) promoter, with or without an enhancer, were transiently transfected into Caco-2 cells (with high CDX2 levels), RKO cells (with low CDX2 levels) and the non-intestinal HeLa cells. Data are shown as the relative luciferase activity compared to the control reporter plasmid, pGL4.10. One-way ANOVA analysis was applied with ± S.E.M (n = 4), **P < 0.01, ***P < 0.001, ****P < 0.0001, n.s = non-significant. The reporter activity was normalized to the co-transfected LacZ plasmid expression.

Our findings have suggested that CDX2 binds to both *ST14* and *SPINT1* enhancers, leading us to speculate if these enhancer regions regulate the transcriptional activity of their corresponding promoters in intestinal cells. To investigate this, a reporter gene assay was conducted by cloning the promoter region of *ST14* and *SPINT1* into a luciferase reporter gene vector together with the corresponding enhancer elements (Fig. 3). For the *ST14* promoter, a 1120 bp sequence was selected, covering the nucleotide position −947 to +173 relative to the TSS (Figure S3). The *SPINT1* promoter was derived from the genomic locus with position −1030 to +27 (1057 bp) relative to the TSS. The cloned plasmids were transiently transfected into Caco-2 cells, and the reporter activity was normalized to the co-transfected LacZ plasmid expression. The *ST14* promoter reporter assay revealed that the *ST14* promoter activity exceeded the activity baseline of the empty pGL4.10 vector, with a significant 27.1-fold stimulation (Fig. 3a). Addition of the *ST14* enhancer together with the promoter increased the *ST14* promoter luciferase activity 6-fold additionally (Fig. 3a). This *ST14* reporter assay was repeated in RKO cells, another colorectal cancer-derived cell line. Unlike the Caco-2 cells, RKO cells do not differentiate into an intestinal-like morphology and have low CDX2 expression^37^. This assay showed that the relative luciferase activity of the *ST14* promoter was 6.8-fold higher than the activity baseline of the empty pGL4.10 vector (Fig. 3a). Interestingly, unlike the Caco-2 cells, the *ST14* luciferase activity seemed to be unaffected by the addition of the *ST14* enhancer to the promoter in RKO cells. Furthermore, the *ST14* reporter constructs were validated in the non-intestinal cervical cancer HeLa cell line devoid of *CDX2*^38^. Neither the *ST14* promoter nor the promoter including the enhancer construct was able to exceed the basal luciferase activity of the empty pGL4.10 reporter vector in HeLa cells (Fig. 3a). In summary, the *ST14* promoter was functional with significant activity in Caco-2 cells and RKO cells. The *ST14* enhancer was only able to stimulate the promoter activity in Caco-2 cells, suggesting that high levels of CDX2 can stimulate the *ST14* enhancer element.

The *SPINT1* reporter assay done in Caco-2 cells revealed that the *SPINT1* enhancer element increased the *SPINT1* promoter activity 5.4-fold (Fig. 3b). In RKO cells, the *SPINT1* promoter luciferase activity was 32.7 times higher than the activity of the empty pGL4.10, and the addition of the *SPINT1* enhancer sequence further stimulated the promoter activity with a significantly 5.7-fold up-regulation (Fig. 3b). In HeLa cells, some *SPINT1* promoter luciferase activity was detected, as compared to the activity of the empty pGL4.10. However, the addition of the *SPINT1* enhancer had no stimulatory effect, suggesting that this enhancer region is intestinal-specific (Fig. 3b). Overall, the *SPINT1* promoter was remarkably active and was stimulated by the putative *SPINT1* enhancer in both Caco-2 cells and RKO cells.

### CDX2 binding sites in the *ST14* and *SPINT1* enhancers

**Figure 4:**
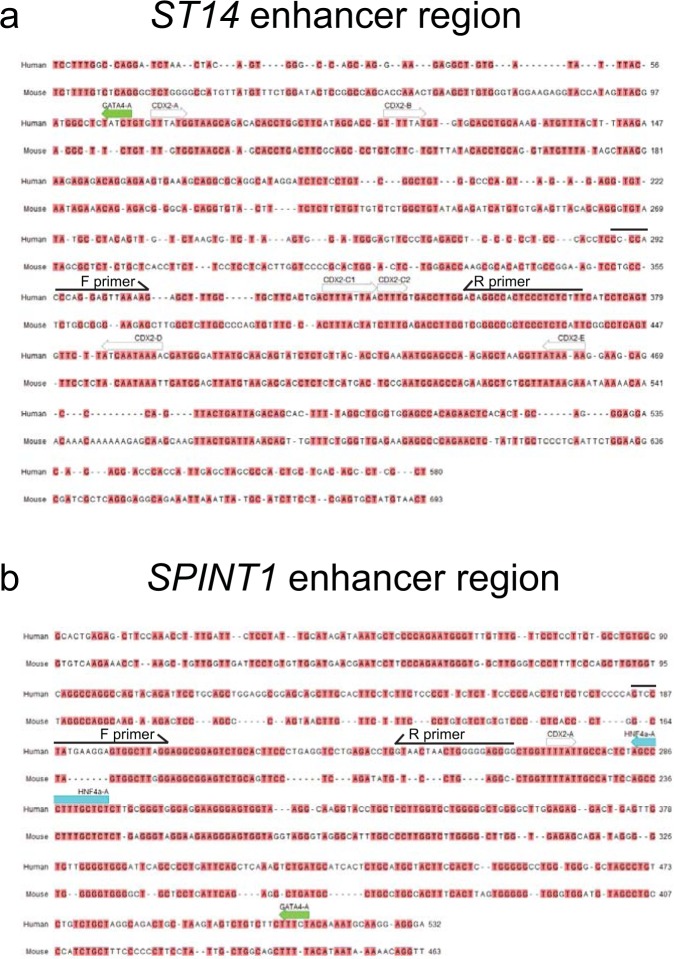
*ST14* and *SPINT1* enhancers contain several predicted CDX2-binding sites. *In silico* sequence analysis of the predicted human *ST14* enhancer, position +21579 to +22159 (580 bp), and of the *SPINT1* enhancer, position +3331 to +3863 (532 bp), relative to the transcriptional start site, shown in (**a** and **b**) respectively. The search for transcription factor binding sites was performed using the Transfac database. Predicted CDX2 binding sites are marked in white, GATA4 binding in green and HNF4α binding in blue. The indicated “F primer” and “R primer” are the forward and reverse primer pairs that have been used to amplify CDX2 bound regions in the ChIP-PCR assay in Fig. 2(c) and (d). The human sequences are aligned to the mouse genome using Clustal W analysis and conserved nucleotides are shown in red.

Based on the CDX2-ChIP enrichment analyses of the putative enhancer regions, the nucleotide sequences of *ST14* (+21579 to +22159 relative to the TSS) and SPINT1 (+3331 to +3863) were next analyzed for possible CDX2 binding sites. Using the transcription factor binding prediction tool, Transfac database, we found five predicted CDX2 binding sites (CDX2-A, CDX2-B, CDX2-C, CDX2-D, and CDX2-E) within the *ST14* enhancer region (Fig. 4a) and one predicted CDX2 binding site (CDX2-A) in the *SPINT1* enhancer element (Fig. 4b). The selected *ST14* enhancer sequence also harbored a single predicted GATA-4 binding site, whereas the *SPINT1* enhancer contained a predicted GATA-4 as well as an HNF4α site. Both GATA-4 and HNF4α are transcription factors known to be involved in the intestinal transcriptional regulatory network^20^. All the strongest CDX2 ChIP binding peaks were observed in the enhancers; however, several predicted CDX2 binding sites were also observed in the promoters of both *ST14* and *SPINT1* (Figure S3 and S4).

**Figure 5:**
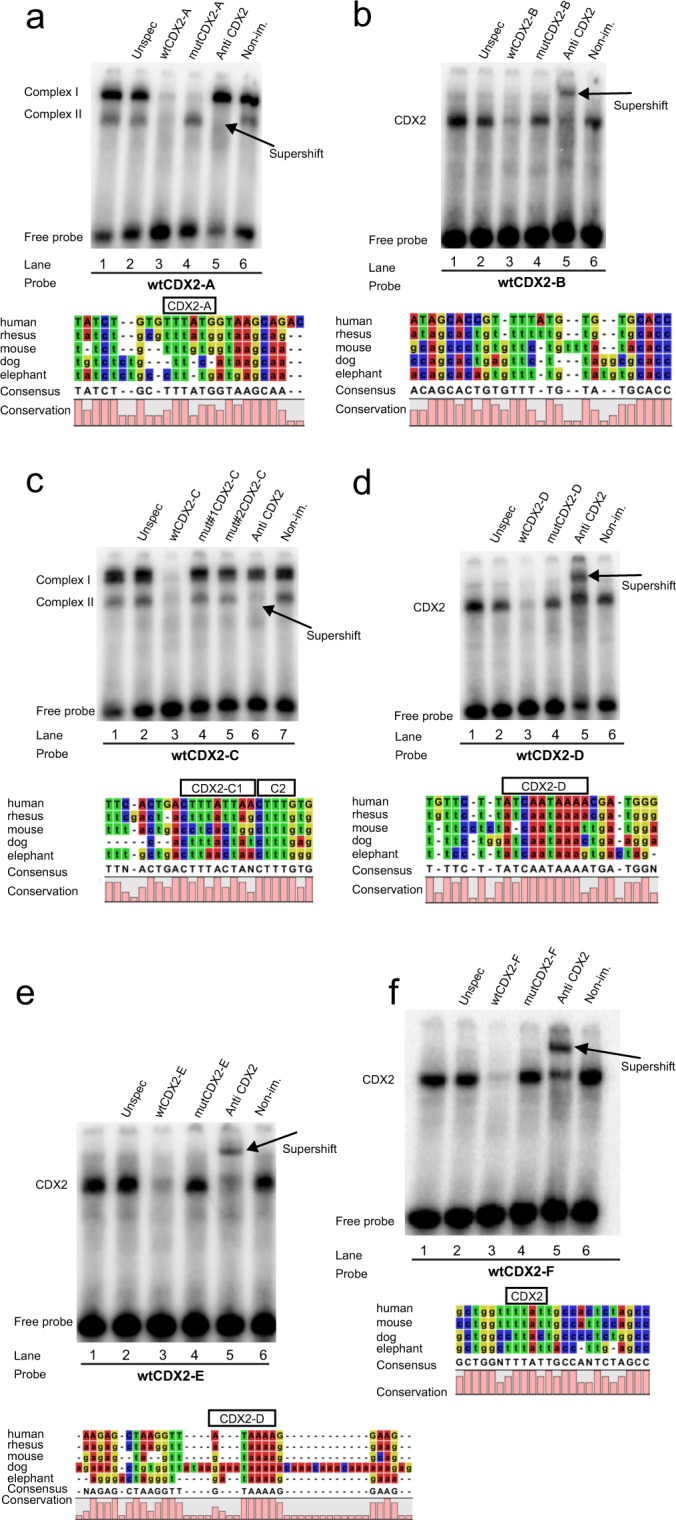
CDX2 binds all the predicted binding-sites within the *ST14* and *SPINT1* enhancers in Caco-2 cells *in vitro*. Gel shift analysis of double-stranded [γ^32^]ATP-labeled oligonucleotides wtCDX2-A (**a**) wtCDX2-B (**b**) wtCDX2-C (**c**) wtCDX2-D (**d**) wtCDX2-E (**e**) covering the putative CDX2 binding sites within the *ST14* enhancer and one within the *SPINT1* enhancer (**f**) [γ^32^] ATP-labeled probes were incubated with Caco-2 nuclear cell extracts, and all resulted in shifted DNA/protein complexes, depicting that binding has taken place (lane 1). Competition by excess of unlabeled unspecific oligonucleotides (lane 2), unlabeled wild-type oligonucleotides (wt) (lane 3) and unlabeled mutated oligonucleotides (mut) (lane 4 (for C lanes 4 and 5)), were used for validating the specificity of the shifted DNA/protein complexes. Addition of anti-human CDX2 antibody was used for the supershift assays (lane 5 (for C lane 6)), and non-immune serum was used as negative control (lane 6 (for C lane 7)). Supershifted complexes are indicated by red arrows. Gels are representative of three independent EMSA assay experiments.

In order to validate physical interaction between CDX2 and the predicted binding sites in the enhancers, electrophoretic mobility shift assays (EMSA) and supershift assays were conducted. Double-stranded probes covering the putative CDX2-binding sites were labeled with [γ^32^]ATP to visualize the physical interaction with proteins from Caco-2 nuclear cell extracts. The probes covering the *ST14* CDX2-B, CDX2-D and CDX2-E (Fig. 5b,d and e respectively) and the probes covering the *SPINT1* CDX2-A (Fig. 5f) all formed one distinct EMSA complex with the nuclear extract (lane 1). These protein/DNA complexes proved to be specific for the probe, as adding a 100-fold excess of unlabeled unspecific oligonucleotide (lane 2) did not compete the interactions significantly. In contrast, the control sample containing excess of unlabeled CDX2 oligonucleotides (lane 3) effectively outcompeted the specific EMSA band. Addition of excess unlabeled oligonucleotide with mutation in the predicted CDX2 binding site did not outcompete the specific EMSA complex, showing that this interaction depends on a specific CDX2 binding motif (lane 4). Finally, addition of CDX2-antibody (lane 5) created a supershift of the probe, confirming that CDX2 is part of the complex, whereas the non-immune serum (lane 6), serving as a negative control, did not affect the complex.

The probe covering the CDX2-A of the *ST14* enhancer formed two specific protein/DNA complexes (complex I and complex II) in the EMSA gel (Fig. 5a, lane 1). Addition of excess unlabeled oligonucleotide, with a mutation in the predicted CDX2-A binding site, competed complex I but not complex II, indicating that formation of complex II depends on the CDX2-A binding site (Fig. 5a, lane 4). Binding of CDX2 to CDX2-A was confirmed, by adding CDX2 specific antibodies, which resulted in a supershift of the complex II, possibly to the same electrophoretic position as complex I (Fig. 5a, lane 5). Complex I is probably formed by another nuclear protein than CDX2.

The probe covering *ST14* CDX2-C contained two potential CDX2 sites (CDX2-C1 and CDX2-C2) and formed two specific protein/DNA complexes (complex I and complex II) in the EMSA gel (Fig. 5c, lane 1). Addition of excess unlabeled wild-type oligonucleotide confirmed the specificity of the probe, by outcompeting both formed complexes (Fig. 5c, lane 3). Addition of unlabeled oligonucleotide, with a mutation in either of the two predicted binding sites, revealed that formation of both complex I and complex II depend on these sites (Fig. 5c, lane 4 and 5). However, only complex II was shifted upon addition of CDX2 antibody (Fig. 5c, lane 6).

All of the predicted CDX2 binding sites within the *ST14* and *SPINT1* enhancer regions were bound by CDX2 in the Caco-2 nuclear cell extracts. These putative binding sites are likely involved in the CDX2-enrichment detected within the *ST14* and *SPINT1* enhancers in Caco-2 cells, shown in Fig. 2.

### *ST14* and *SPINT1* promoters and enhancers are affected by CDX2 levels in intestinal cells

**Figure 6:**
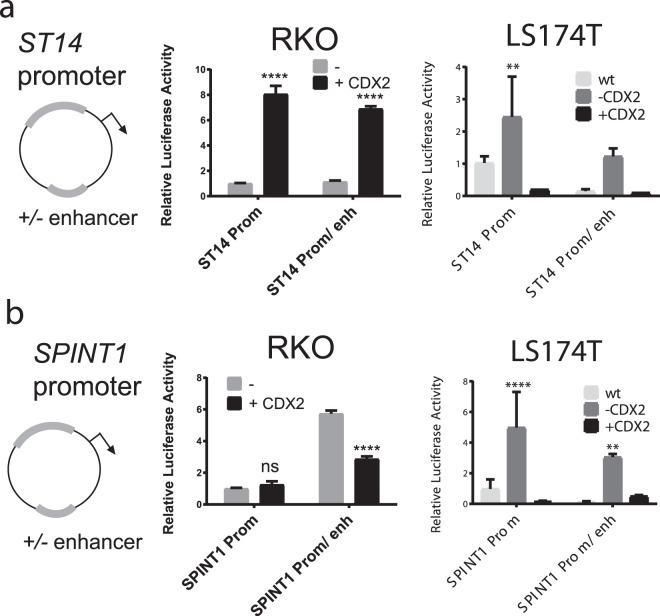
CDX2 regulates *ST14* and *SPINT1* reporter constructs in intestinal cells. *ST14* (**a**) and *SPINT1* (**b**) reporter plasmids were analyzed in intestinal RKO and LS174T cells. RKO cells were transiently transfected with or without overexpression of CDX2. For the LS174T cells, wild type cells were compared to cells with doxycycline-regulated CDX2 levels. (**a**) The *ST14* promoter and promoter/enhancer activity were stimulated by CDX2 in RKO cells. In LS174T cells, the opposite is observed where CDX2 knock-out (−CDX2) resulted in increase of *ST14* promoter and enhancer activity. (**b**) CDX2 did not affect *SPINT1* promoter activity, but reduced *SPINT1* enhancer activity. In LS174T cells loss of CDX2 (−CDX2) resulted in increased *SPINT1* and enhancer activity. The reporter activity was normalized to the co-transfected LacZ plasmid expression, (mean values presented as S.E.M, two-way ANOVA analysis) n = 4 (****P < 0.0001).

The higher *SPINT1* promoter/enhancer activity observed in the RKO cells with low CDX2 expression, as compared to Caco-2 cells, could be explained by the high CDX2 levels in Caco-2 cells having an inhibitory effect on the *SPINT1* reporter activity. To clarify the regulation by CDX2, we next analyzed the *ST14* and *SPINT1* reporter construct in both RKO cells and in LS174T cells with manipulated levels of CDX2. RKO cells transiently transfected with a CDX2-expressing plasmid showed that overexpression of *CDX2* had a stimulatory effect on the *ST14* promoter luciferase activity (Fig. 6a). This CDX2-mediated stimulation of the promoter can be explained by the observation that the promoter region contains *in silico* predicted CDX2 binding-sites (Figure S3). The *ST14* enhancer construct did not increase the promoter activity any further upon CDX2 overexpression in RKO cells (Fig. 6a). However, it is possible that the high CDX2 levels saturate the ST14 promoter/enhancer activity, or that the RKO cells lack additional transcription factor(s) required for the optimal *ST14* enhancer activity. Surprisingly, in LS174T cells CDX2 displays an inhibitory role towards the *ST14* promoter and enhancer (Fig. 6a). Both the promoter and enhancer activities are increased upon loss of CDX2 (−CDX2). The result does not correlate with the increase of the *ST14* mRNA upon gain of CDX2 expression seen in Fig. 1. We suggest that that this apparent discrepancy might be due to the endogenously regulatory mechanisms and the chromatin structure that might not be faithfully reproduced using transfected reporter constructs. In conclusion, this indicates that CDX2 can exhibit either enhancing or repressive regulatory activity towards *ST14* in a cell-type specific manner.

**Figure 7:**
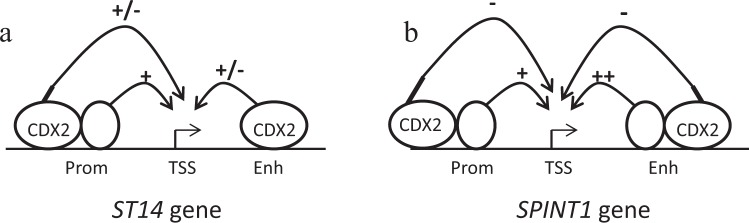
Model for the transcriptional regulation of *ST14* and *SPINT1* genes in intestinal cells. In RKO cells, which have low levels of CDX2, the *ST14* promoter is active and can be stimulated by overexpression of CDX2. However, the enhancer is inactive in RKO cells, probably due to low levels of CDX2 and/or lack of additional co-factors. In Caco-2 cells, which have high levels of CDX2, both the *ST14* promoter and enhancer are active. In LS174T (+Dox) cells, re-activation of CDX2 expression (**a**) leads to stimulation of *ST14* gene expression, probably due to increased CDX2-dependent promoter and enhancer activity at the endogenously level. However, in transfection experiments CDX2 has an inhibitory regulatory activity on *ST14* reporter constructs in LS174T cells. At the *SPINT1* gene, both the promoter and enhancer are active in Caco-2 and RKO cells, and the promoter activity is not affected by CDX2 overexpression in RKO cells. However, the enhancer activity is reduced in RKO cells which overexpress CDX2, suggesting an inhibitory effect of CDX2 at the *SPINT1* enhancer. Similarly, re-activation of CDX2 expression in LS174T (+Dox) cells (**b**) leads to inhibition of *SPINT1* gene expression, probably due to CDX2-mediated repression at the enhancer. *SPINT1* reporter experiments in LS174T cells confirm that CDX2 acts as an inhibitor at the enhancer site but also in the promoter region. Open circles indicate transcription factors. Prom, promoter; TSS, transcription start site; Enh, enhancer.

In RKO cells, overexpression of CDX2 did not affect the *SPINT1* promoter luciferase activity but had an inhibitory effect on the *SPINT1* enhancer activity (Fig. 6b). The high *SPINT1* enhancer activity in the control RKO cells is proposed to be driven by a CDX2-independent regulatory mechanism which CDX2 can inhibit. In LS174T cells, loss of CDX2 resulted in an increase of both *SPINT1* promoter and enhancer activity (Fig. 6b). Our *in silico* analysis predicted a single CDX2 binding-site in the *SPINT1* promoter which could be functional as a repressive site in LS174T cells. The *SPINT1* reporter constructs were repressed in the CDX2 gain of function condition (+CDX2) compared to the loss of function (−CDX2) condition showing that indeed CDX2 is a repressor of *SPINT1*. The mechanisms regulating *SPINT1* transcription in intestinal cells, which CDX2 inhibits, still remain to be clarified. Overall, these findings suggest that CDX2 can have both stimulatory and repressive effects on the *ST14* enhancer/promoter and an inhibitory effect on *SPINT1* in intestinal cells (Fig. 7).

## Discussion

The identification of HAI-1 and HAI-2 has clearly helped to understand how the enzymatic activity and the oncogenic potential of matriptase is tightly regulated and controlled. Matriptase and its inhibitors have been well elucidated at the protein level; however, the regulatory mechanisms maintaining their gene expression are less clearly understood. The stoichiometric balance of matriptase and its inhibitors is key, as a shift towards increased matriptase expression and its enzymatic activity has oncogenic potential. Matriptase and SPINT1 are normally expressed in the epithelium of both small intestine and colon where they are involved in epithelial barrier integrity and are also suggested to be involved in cellular turnover of the gut^3,39–41^. Other examples have demonstrated the importance of protease/inhibitor balance in health and disease state. The ratio of matrix metalloproteinase-2 (MMP2) and its inhibitor TIMP2 has been found to be imbalanced in hepatic fibrosis whereas MMP-9/TIMP-1 ratio is dysregulated in liver metastasis^42,43^. The matriptase/HAI-1 mRNA ratio has previously been demonstrated to be dysregulated in colorectal cancer tissue^19^. Both *ST14* and *SPINT1* gene expression levels were reported to be lower during colorectal carcinogenesis compared to normal healthy patient tissue samples. Dysregulation in the network of transcription factors is likely to take part in the altered *ST14/SPINT1* expression pattern. In this presented study, we have discovered a role for the intestinal-specific transcription factor, CDX2 in regulating both *ST14* and *SPINT1* through an intestinal-specific enhancer regulatory element. Using intestinal cell line studies, we dissected genetic mechanisms *in vitro* and demonstrated that CDX is not required for *ST14* and *SPINT1* basal gene expressions but the transcription factor has the ability to alter the balance in the *ST14/SPINT1* mRNA ratio. The identified CDX2-bound enhancer elements for *ST14* and *SPINT1* were shown to be functional in intestinal epithelial cells. CDX2 has previously been demonstrated to affect intestinal enhancer activity of *APC* and *AXIN2* from the Wnt pathway, which is known to be dysregulated in colon cancer^44^. Our observation that induced CDX2 expression in LS174T cells promotes an increased *ST14/SPINT1* ratio does not correlate with the reported expression pattern of decreased CDX2 levels and increased *ST14/SPINT1* levels during colon cancer progression. However, our reporter experiments show that CDX2 can display both enhancing and repressive regulation of *ST14* regulatory elements depending on the intestinal cell line. It is likely that CDX2 can either enhance or repress *ST14* depending on the available network of transcription factors and the status of the epigenetic control of the regulatory elements. However, whether dysregulated CDX2 levels correlate with *ST14* and *SPINT1* in clinical colorectal cancer specimen needs to be clarified. CDX2 is well-known for its positive regulation of transcription in the intestine, but its role as an intestinal transcriptional repressor is not well-characterized. Beside our observation of CDX2 being a repressor of *SPINT1*, it has previously been demonstrated that CDX2 represses insulin-like growth factor binding protein-3 (IGFBP-3) in the human colon cell line, Lovo^45^. CDX2 has moreover been demonstrated to be a repressor of Oct4 during blastocyst development by interacting with the chromatin remodeling factor, Brahma related gene 1(BRG1)^46,47^. It is possible the CDX2-mediated repression of *SPINT1* and *ST14* involves the recruitment of BRG1 which is also expressed in the intestine^48^. The basal *SPINT1* mRNA expression and the synergistic activity of the enhancer and promoter do not seem to be CDX2 dependent, and the transcriptional mechanisms required for *SPINT1* expression in the intestine still remains to be elucidated. Previous promoter analysis of *SPINT1* has demonstrated SP1 and Egr1–3 binding-sites^49,50^ which raises the possibility that these factors could be involved in regulating *SPINT1* expression in the intestine.

Our identified functional enhancers for *ST14* and *SPINT1* are covered in H3K4me2 layers in Caco-2 cells which indicates that changes in epigenetic histone modifications could also potentially be involved in regulating *ST14* and *SPINT1* in health and disease state. A recently interesting study has demonstrated that CDX2 are also involved in mediated anti-repressive gene regulation by excluding H3K27me3 at enhancer sites thus de-repressing epigenetic silencing^51^. Gene-regulatory enhancer elements and their epigenetic states are considered to be a driving force of spatial and temporal gene expression pattern through development and specification of tissues and cell fate^52^. Recently, genomic high-occupancy target (HOT) regions and super-enhancers (SEs) have got increased attention, as they are believed to be involved in regulating the gene expression pattern of the developing disease state and can be sensitive towards oncogenic signaling networks^53,54^. CDX2 has also been demonstrated to affect epigenetic chromatin modifications and control occupancy of other intestinal transcription factors^55,56^. Our reporter studies showed that CDX2 directly interacts with the *ST14* and *SPINT1* enhancer sites; however, it is also possible that CDX2 has secondary effects by remodeling the chromatin and accessibility for other transcription factors at these sites. We did observe binding-enrichment of the transcription factor HNF4alpha in the *ST14* and *SPINT1* enhancer region (Figure S1 and S2). As HNF4a is known to regulate intestinal gene expression in concert with CDX2^22^, it is likely that HNF4alpha is also involved in regulating *ST14* and *SPINT1*.

The link between CDX2 and matriptase is further interesting as they both affect intestinal inflammation and colitis-associated cancer. Patients suffering from Crohn’s disease and Ulcerative Colitis have been reported to have reduced *ST14* expression^5^ and mouse studies have shown that matriptase has a suppressive and protective role towards colitis and colitis-associated carcinogenesis^5,11^. Matriptase has a critical function in regulating the intestinal epithelial barrier and integrity including regulation of tight junction protein, Claudin-2^4,39,57^. Likewise, CDX2 is also involved in regulation of expression of Claudin genes^58–63^ and is involved in inflammatory bowel disease and cancer^64–69^.

In summary, we have identified CDX2 as a regulator of both *ST14* and *SPINT1* at the transcriptional level in intestinal cells. As both CDX2 and matriptase have clinical relevance, we suggest that future research might benefit from this link between the transcription factor and the protease/inhibitor system in relation to intestinal disorders.

### Experimental procedures

#### Reporter plasmid construction

The primers for cloning listed in Table S1 were used to amplify the promoters and enhancers from human genomic DNA (Roche Diagnostics). The sequence positions are relative to the transcriptional start site; *ST14* promoter position −947 to +173 (1120 bp) (GenBankTM number NM_021978), *ST14* enhancer position +21579 to +22159 (580 bp), *SPINT1* promoter position −1030 to +27 (1057 bp) (GenBankTM number NM_181642) and *SPINT1* enhancer position +3331 to +3863 (532 bp). The Gateway Technology (Invitrogen) was used to clone the selected promoter sequences into a modified pGL4.10 (Promega) firefly luciferase reporter vector, as previously described^36^. The enhancer elements were cloned into their corresponding promoter plasmids using the BamHI or SalI site in the pGL4.10 vector. All the plasmid constructs were sequence verified (Eurofins MWG, Germany).

#### Cell Culturing

The human colon cancer cell lines, Caco-2, RKO and the cervical HeLa cells (American Tissue Type Culture Collection) were grown in Dulbecco’s Modified Eagle’s Medium (DMEM) containing 4.5 g/L glucose, L-glutamine and supplemented with 10% heat-inactivated foetal bovine serum (FBS), 100 U/ml penicillin and 100 U/ml streptomycin. Cells were grown in T175 flasks (Nunc) at 37 °C in a humid atmosphere with 5% CO_2_. LS174T-derived cell lines were cultured in a 1:1 mixture of DMEM and Ham’s nutrient mixture F12, containing 10% FBS and 1% glutamine. For Tet-On experiments with the inducible LS174T modified cells, cells were treated by addition of either 0 or 2–12 ng doxycycline/well (using 24 well system, Costar) to the media every 24 hours until harvest as previously described^32^. Briefly, the isogenic CDX2 knockout and doxycycline induced rescue model was designed by integration of the pCDX2-pCMV-TET3G-ObLiGaRe donor vector into LS174T cells. The pCMV-Tet3G were targeted to disrupt and knock-out the endogenously Exon1 of the CDX2 locus whereas the doxycycline inducible CDX2 expression construct was integrated at the safe genomic AAVS1 locus.

#### Transfection and Luciferase reporter assay

Caco-2 cells and RKO cells were seeded in 24 well plates (Costar) at a density of 5 × 10^4^ cells/well whereas HeLa cells were seeded at a density of 4 × 10^4^ cells/well. The cells were transfected the following day at an approximately 60–80% cell confluence. A non-commercial transfection reagent termed PEI25 was used as follows: Polyethyleneimine (Alfa Aesar, cat#43896) dissolved in milliQ water and calibrated to pH7 using 4 M HCl. The use of PEI25 as a transfection reagent was validated by comparing with previously used commercial transfection reagent. PEI25 has an acceptable transfection efficiency, and it does not have an impact on the transfection results due to the change of transfection reagent. Each transfection experiment contained 0.2 µg of pGL4.10 reporter plasmid construct, 0.1 µg pCMV-lacZ (internal transfection control) and pBluescript II SK+ plasmid added to correct the total amount of DNA to 1.2 µg. For CDX2 overexpression, 0.1 µg of either pCMV-CDX3 (CDX2 hamster homologue-expressing vector) or pcDNA3.1+ (CMV-driven control vector) was used. The DNA was mixed with 2 µM PEI25, and the experiment was divided into four replicates. Two days after transfection, the cells were harvested and the luciferase and β-galactosidase activity were measured on a luminometer (Berthold, Lumat LB9501), using the Dual Light assay (Tropix). The luciferase activity was normalized to the β-galactosidase activity.

#### Quantitative RT-PCR

For measuring gene expression, total RNA was harvested from cultured cells using the RNeasy Kit (Qiagen) according to the manufacturer’s instructions. The concentration of RNA was measured, and approximately 250–300 ng RNA was converted into cDNA using High-Capacity cDNA Archive Kit (Applied Biosystems). The TaqMan system was applied for gene expression quantification, using the primers and probes for target genes listed in Table S4. The reactions were run on an ABI7300 sequence detection system (Applied Biosystems) using Universal PCR Master Mix (Applied Biosystems). The PCR amplification program was 95 °C for 10 minutes followed by 40 cycles of 95 °C for 15 seconds and 65 °C for 15 seconds. Negative controls (no reverse-transcriptase reaction) as well as positive controls were included in all experiments. The Step-One Software (Applied Biosystems) was used to validate the results and the following baseline threshold settings for the specific target genes were set as previously described^19^: ST14 (0.2), SPINT1 (0.025) and β-Actin (0.2). Quantification of relative gene expression was normalized to β-Actin levels and calculated using the standard comparative Ct method.

#### Electrophoretic Mobility Shift Assay

Electrophoretic Mobility Shift Assay (EMSA) was conducted to verify DNA-protein binding. Oligonucleotides used in this experiment, listed in Table S2, were designed to cover putative CDX2-binding sites based on prediction by an *in silico* analysis using Transfac Professional 12.1 database. The probes were formed by annealing the sense and antisense oligos followed by an 5′-end-labelling with [γ-^32^P] ATP (Perkin Elmer), using T4 polynucleotide kinase (Fermentas). Nuclear extracts were prepared as previously described^70^. The EMSA protein/DNA binding reaction contained 2 μl of 10 day differentiated Caco-2 nuclear extracts, 3 μl dialysis buffer (25 mM Hepes pH 7.6, 40 nM KCl, 0.1 mM EDTA and 10% glycerol), 10 μl Gelshift buffer (25 mM Tris-HCl pH 7.5, 5 mM MgCl2, 60 mM KCl, 0.5 mM EDTA, 5% Ficoll 400, 2.5% glycerol, 1 mM DTT and protease inhibitors), 0.5 μl dI-dC (homopolymer of deoxyinosine and deoxycytidine). For the competition assay, a 100-molar excess of unlabelled unspecific, wild-type or CDX2-binding site-mutated oligonucleotide was added to the reaction mixture. For supershift assays, 1 µl of CDX2-antibody (BioGenex, cat#MU392A-UC) or 1 µl of non-immune serum (normal rabbit serum, Santa Cruz Biotechnology, SC-2338) was added. The reaction mix was incubated on ice for 20 minutes and then 1 μl of radiolabeled probe was added followed by a further incubation for 20 minutes on ice. Finally, 2 μl of Gelshift loading buffer (10% glycerol, 0,2% Bromphenol blue and 0,5xTris-borate-EDTA buffer (2xTBE, 44.5 mM Tris-HCL pH 8.0, 44.5 mM boric acid and 1 mM EDTA)) was added to the binding reaction mixture. The protein/DNA complexes were resolved on a 5% non-denaturing polyacrylamide gel which afterward was dried on a slab gel dryer SGD4050 (Savant) before exposure using a phosphor-image screen. The screen was scanned on a Storm 840 scanner (Molecular Dynamics) and the image was analysed using the Image-Quant Software version 5.2.

#### Chromatin Immunoprecipitation (ChIP) analysis

Caco-2 cells were grown for 5–6 days after confluence before harvest for the ChIP experiment. Cross-linking of protein/DNA, sonication, immunoprecipitation and DNA purification was done as previously described^21^. Sonication was verified for yielding DNA fragments of approximately 100–500 bp. The immunoprecipitations were performed in four replicates with either CDX2 antibody (BioGenex, Mu329A-UC) or HA (hemagglutinin) antibody (Santa Cruz Biotech, sc-805), using Dynabeads Protein A/G beads. From each replicate, 1% was removed before immunoprecipitation and used as input control. Analysis of immunoprecipitated DNA enrichments was performed with qPCR using the primers listed in Table S3 and SYBR Green I Master Mix (Roche). The primers amplifying the CDX2-bound region for ChIP-PCR assay were designed to cover CDX2-enriched enhancer peaks observed in the CDX2 ChIP-seq tracks. The samples were run on a LightCycler 480 PCR machine (Roche Diagnostics), using LightCycler Software version 1.5 (Roche Diagnostics). The relative ChIP-DNA enrichments were calculated as previously reported^71^.

#### Bioinformatics

The genomic sequence tracks presented in this study derive from previously published and available data; Caco-2 CAGE-tracks (NCBI GEO data: accession No. GSE54074)^72^, Caco-2 CDX2 ChIP tracks^21^ (available upon request), Caco-2 NNF4a- and H3K4me2 CHIP (NCBI GEO data: accession No GSE23436), LS174T CDX2 ChIP (NCBI GEO data: accession No GSE97273). The sequencing data were mapped to hg19 and the CisGenome version2^73^ software was used to analyze the data. Tracks were uploaded into UCSC Genomebrowser^74^ for visualization images precented in this study.

#### Statistical analysis

Both Students T-test (with mean as standard deviation, S.D.) and ANOVA test (presented with standard error mean, S.E.M.) were used on data presented in this study. Graphs and calculated statistical significance were created using the GraphPad Prism 5 software.

## Electronic supplementary material

Supplmentary Information
