# Supplementary material for: Intestinal regulation of suppression of tumorigenicity 14 (ST14) and serine peptidase inhibitor, Kunitz type -1 (SPINT1) by transcription factor CDX2
Source: Sci Rep. 2018 Aug 7;8:11813. doi: 10.1038/s41598-018-30216-z (PMC6081401; doi:10.1038/s41598-018-30216-z)

Intestinal regulation of *suppression of tumorigenicity 14 (ST14)* and *serine peptidase inhibitor, Kunitz type -1 (SPINT1)* by transcription factor CDX2

E. Thomas Danielsen, Anders Krüger Olsen, Mehmet Coskun, Annika W. Nonboe, Sylvester Larsen, Katja Dahlgaard[1,] Eric Paul Bennett, Cathy Mitchelmore, Lotte Katrine Vogel and Jesper Thorvald Troelsen

Supporting Information

Name	Sequence (5’-3’)
st14 prom.	F: AACAGTGGAAAATGGGCAAG<br>R: GGTCTCACAGGCGTCGTC
st14 enh.	F: GGATCCTCCTTTGGCCAGGATCTAAC<br>R: GGATCCAGCGAGGCTGTCAGCAGT
SPINT1 prom.	F: TGGAGGATGTTGCAGTTCAG<br>R: CTTCTCCCCCTGGTTTCTG
SPINT1 enh.	F: GTCGACGCACTGAGAGCTTCCAAACC<br>R: GTCGACTCCCTCCTTGCATTTTGTAGA

Table S1: Primers targeting genomic DNA amplification used for cloning and construction of luciferase reporter plasmids.

Name	Sequence (5’-3’)
ST14 enh_wtCDX2-A	F: GATCCTATCTGTGTTTATGGTAAGCAGACG<br>R: TCGACGTCTGCTTACCATAAACACAGATAG
ST14 enh_mutCDX2-A	F: GATCCTATCTGTGCATATGGTAAGCAGACG<br>R: TCGACGTCTGCTTACCATATGCACAGATAG
ST14 enh_wtCDX2-B	F: GATCCATAGCACCGTTTTATGTGTGCACCG<br>R: TCGACGGTGCACACATAAAACGGTGCTATG
ST14 enh_mutCDX2-B	F: GATCCATAGCACCGCATATGGTGTGCACCG<br>R: TCGACGGTGCACACCATATGCGGTGCTATG
ST14 enh_wtCDX2-C	F: GATCCTTCACTGACTTTATTAACTTTGTGG<br>R: TCGACCACAAAGTTAATAAAGTCAGTGAAG
ST14 enh_mutCDX2-C_#1	F: GATCCTTCACTGACTTCATATGCTTTGTGG<br>R: TCGACCACAAAGCATATGAAGTCAGTGAAG
ST14 enh_mutCDX2-C_#2	F: GATCCTTCACTGACTTCATATGCTCTGTGG<br>R: TCGACCACAGAGCATATGAAGTCAGTGAAG
ST14 enh_wtCDX2-D	F: GATCCTGTTCTTATCAATAAAACGATGGGG<br>R: TCGACCCCATCGTTTTATTGATAAGAACAG
ST14 enh_mutCDX2-D	F: GATCCTGTTCTTCATATGAAAACGATGGGG<br>R: TCGACCCCATCGTTTTCATATGAAGAACACCTAG
ST14 enh_wtCDX2-E	F: GATCCAAGAGCTAAGGTTATAAAAGGAAGG<br>R: TCGACCTTCCTTTTATAACCTTAGCTCTTG
ST14 enh_mutCDX2-E	F: GATCCAAGAGCTAAGGTTCATATGGGAAGG<br>R: TCGACCTTCCCATATGAACCTTAGCTCTTG
SPINT-1 enh_wtCDX2	F: GCTGGTTTTATTGCCACTCTAGCC<br>R: GGGCTAGAGTGGCAATAAAACCAG
SPINT-1 enh_mutCDX2	F: TGGGGGAGGGGCTGGCATATGTGCCACTCTAGCCCT<br>R: GGGCTAGAGTGGCACATATGCCAGCCCCTCCCCCAG
*Unspecific	F: AACGTAGCTGATCGAATCGGTTAC<br>R: AGTAACCGATTCGATCAGCTACGT

Table S2: Oligonucleotides used for EMSA, gel shift assay. Mutations introduced in CDX2 predicted binding sites are underlined. *The sequences for the Unspecific oligonucleotides has previously been demonstrated not to bind CDX2[1].

Name	Sequence (5’-3’)	Amplicon (bp)
ST14 enh.	F: CCCCACCCAGGAGTTAAAAG<br>R: AAAGAGAGGGAGTGGCCTGT	82
SPINT1 enh.	F:GTCCTATGAAGGAGTGGCTTAGG<br>R: CCCCTCCCCCAGTTAGTTAC	80
HEPH prom.	F: AGCAGAGGCCTTATCCCTTC<br>R: GCTGAGATCCAAGTCCAAGC	65

Table S3: Primers used for ChIP-qPCR analysis.

Name	Sequence (5’-3’)	Amplicon (bp)
ST14	F: GCGCTCCCTGAAGTCCTTT<br>R: GTCCTGGGTCCTCTGTACTGTTTT<br>Probe: FAM-TCACCTCAGTGGTGGCTTTCCCCA-BHQ	56
SPINT1	F: CGCGGCATCTCCAAGAAG<br>R: GAACACTGCGACAGCCATCTC<br>Probe:FAM-AAATCCCCATTCCCAGCACAGGCTC-BHQ.	90
β-Actin	Pre-developed and commercially available probe/primers, part. no.4310881E, Applied Biosystems	

Table S4: Primers and probes used for RT-qPCR analysis.

Figure legends

Figure S1. Previous published ChIP-seq tracks[2] reveal that the *ST14* enhancer (marked with red box) is enriched in both CDX2 and HNF4A binding in open chromatin region covered by H3K4me2 in Caco-2 cells. Image obtained from UCSC Genome Browser[3].

Figure S2. Additional ChIP-seq tracks from Caco-2 cells[2] shows that the investigated *SPINT1* enhancer (marked with red box) has binding peaks of H3K4me, CDX2 and HNF4A. Image is extracted from the UCSC Genome Browser[3].

Figure S3. *In silico* analysis of the *ST14* promoter. Clustal W alignment of the human *ST14* promoter sequence (position -947 to +173 (1120 bp) relative to the transcriptional start site) with the mouse sequence. Conserved positions are marked with red. Putative binding sites (obtained from Transfac software) are marked with arrows: CDX2 (white), Sp1 (purple) and GATA4 (green). The graphical view was created with the software, CLC Main Workbench version 6.

Figure S4. *In silico* analysis of the *SPINT1* promoter. Clustal W alignment of the human *SPINT1* promoter sequence (-1030 to +27 (1057 bp) relative to the transcriptional start site (TSS)) with the mouse sequence. Putative binding sites are marked with arrows: CDX2 (white), Sp1 (purple) and GATA4 (green)

List of references

1. Jorgensen, S., Coskun, M., Homburg, K. M., Pedersen, O. B. & Troelsen, J. T. HOXB4 Gene Expression Is Regulated by CDX2 in Intestinal Epithelial Cells. *PloS one* **11**, e0164555, doi:10.1371/journal.pone.0164555 (2016).
2. Verzi, M. P. *et al.* Differentiation-specific histone modifications reveal dynamic chromatin interactions and partners for the intestinal transcription factor CDX2. *Developmental cell* **19**, 713-726, doi:10.1016/j.devcel.2010.10.006 (2010).
3. Tyner, C. *et al.* The UCSC Genome Browser database: 2017 update. *Nucleic acids research* **45**, D626-D634, doi:10.1093/nar/gkw1134 (2017).

Figure S1

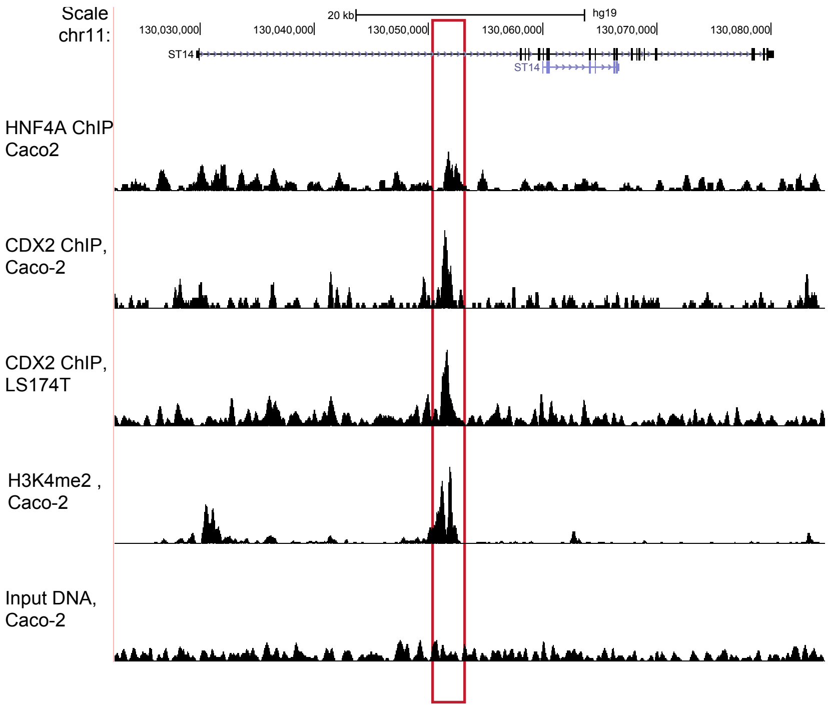

Scale
chr11:
20 kb
hg19
130,030,000
130,040,000
130,050,000
130,060,000
130,070,000
130,080,000
ST14
ST14
HNF4A ChIP
Caco2
CDX2 ChIP,
Caco-2
CDX2 ChIP,
LS174T
H3K4me2 ,
Caco-2
Input DNA,
Caco-2

Figure S2

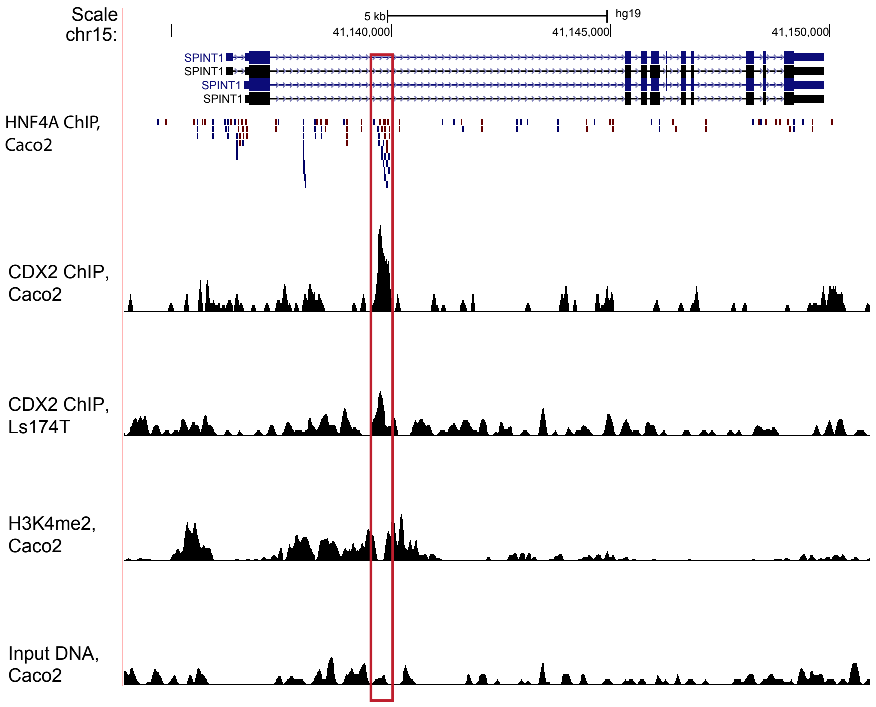
Scale
chr15:
5 kb
hg19
41,140,000
41,145,000
41,150,000
SPINT1
SPINT1
SPINT1
SPINT1
HNF4A ChIP, Caco2
CDX2 ChIP, Caco2
CDX2 ChIP, Ls174T
H3K4me2, Caco2
Input DNA, Caco2

Figure S3

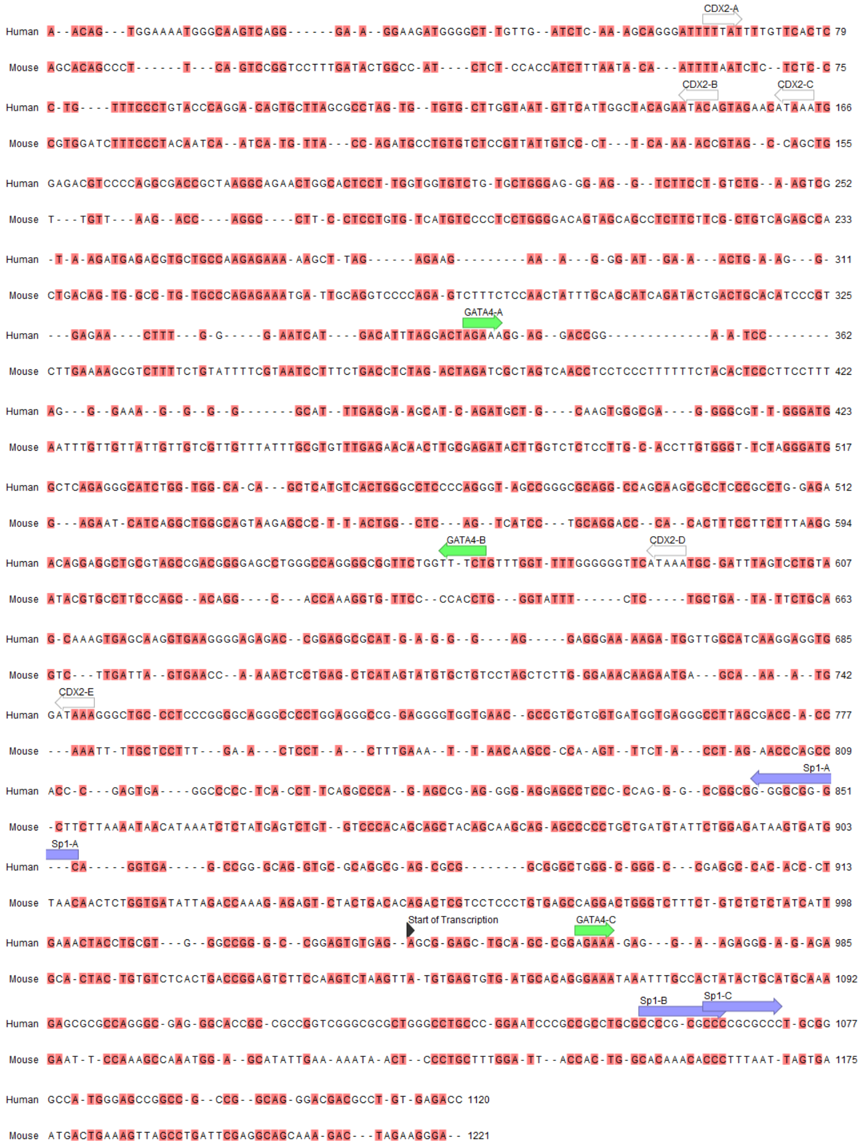
CDX2-A
CDX2-B
CDX2-C
GATA4-A
GATA4-B
CDX2-D
CDX2-E
Sp1-A
Start of Transcription
GATA4-C
Sp1-B
Sp1-C

Figure S4

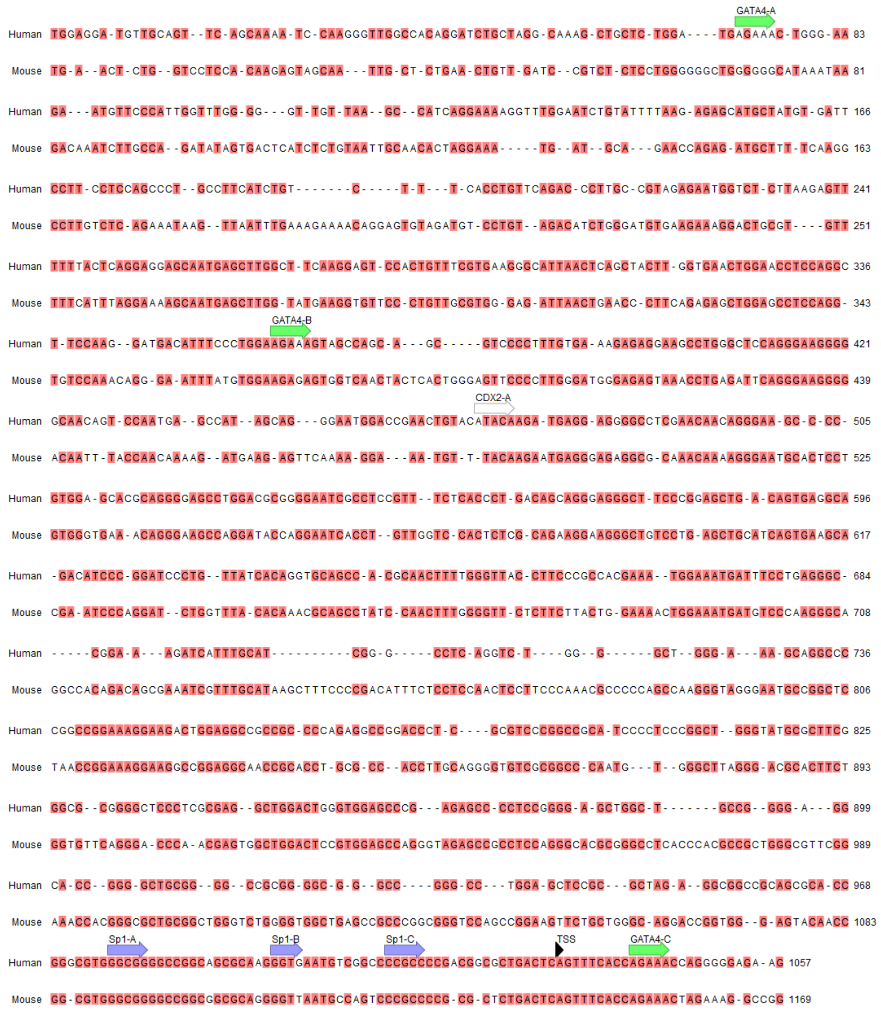

Supplement: Supplementary file 1 — Supplmentary Information [file 41598_2018_30216_MOESM1_ESM.pdf]
